# Chronic Pulmonary Aspergillosis Complicating Bronchial Atresia

**DOI:** 10.1155/2014/208963

**Published:** 2014-12-22

**Authors:** Mazen O. Al-Qadi, Dereddi Raja S. Reddy, Brandon T. Larsen, Vivek N. Iyer

**Affiliations:** ^1^Division of Pulmonary and Critical Care Medicine, Brown University, Providence, RI 02903, USA; ^2^Division of Pulmonary and Critical Care Medicine, Mayo Clinic, Rochester, MN 55905, USA; ^3^Department of Pathology, University of Arizona Medical Center, Tucson, AZ 85724, USA

## Abstract

Bronchial atresia is a rare pulmonary developmental anomaly characterized by the presence of a focal obliteration of a segmental or lobar bronchial lumen. The lung distal to the atretic bronchus is typically emphysematous along with the presence of mucus filled ectatic bronchi (mucoceles). BA is usually asymptomatic but pulmonary infections can rarely develop in the emphysematous lung distal to the atretic bronchus. We present a unique case of chronic pulmonary aspergillosis (CPA) in a patient with BA with no evidence of immune dysfunction. The patient was treated initially with voriconazole and subsequently underwent surgical excision of the involved area. On follow-up, she has done extremely well with no evidence for recurrence. In summary, we describe the first case of chronic pulmonary aspergillosis in an immunocompetent patient with bronchial atresia.

## 1. Case

**Figure 1:**
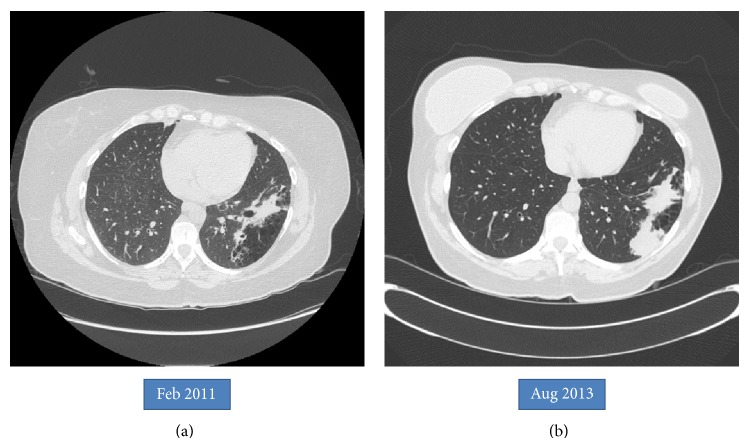
(a) Bronchial narrowing with mucous plugging, surrounded by multiple areas of lucency in the left lower lobe consistent with bronchial atresia. (b) Interval development of a mass-like consolidation after a follow-up period of 30 months.

**Figure 2:**
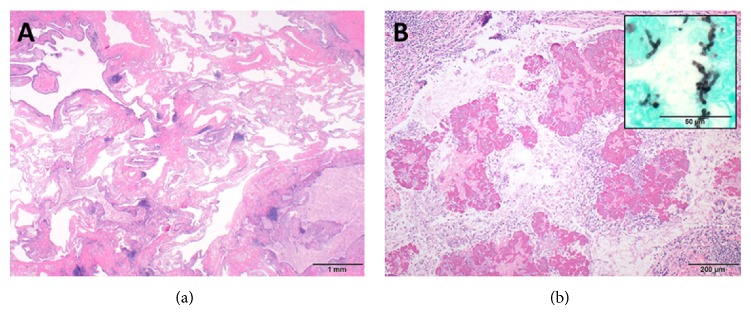
(a) At scanning magnification (20x; hematoxylin and eosin), marked emphysema is present in association with patchy fibrosis and irregular and markedly ectatic bronchioles filled with inspissated debris, consistent with CLH. (b) At medium power (100x; hematoxylin and eosin), amorphous and partially calcified masses fill ectatic bronchioles, accompanied by numerous branching hyphae consistent with* Aspergillus* (inset; 600x; Gomori methenamine silver), findings that are diagnostic of aspergilloma.

An asymptomatic 54-year-old woman was found to have an abnormal chest X-ray during preoperative screening for a right breast adenocarcinoma. A CT chest confirmed the presence of BA along with hyperinflation in the left upper lobe (apicoposterior segment) and left lower lobe (superior and lateral basal segments). The hyperinflated areas in the left upper lobe showed some linear scars whereas the lower lobe showed an area of irregular consolidation (Figure 1). Given the recent diagnosis of breast adenocarcinoma, it was elected to follow the patient with serial chest CT scans to exclude infection or malignancy. The area of consolidation remained unchanged on follow-up chest CT scans obtained 6 and 18 months later. A follow-up scan performed at 30 months showed increased consolidation with a more rounded, mass-like appearance in the lower lobe; however, she remained asymptomatic. A percutaneous CT guided needle biopsy was negative for malignancy, but cultures grew* Aspergillus fumigatus*. Oral voriconazole was started for presumed CPA with a possible aspergilloma. After completing a 12-week course of voriconazole, she underwent an uneventful left lower lobectomy. Histologic examination showed inspissated masses of mucus and fungal hyphae morphologically consistent with* Aspergillus*, filling emphysematous and fibrotic cystic spaces and markedly ectatic bronchioles, confirming a diagnosis of CPA with aspergilloma in the setting of BA and lung hyperinflation (Figure 2). After surgery, she received 3 months of additional voriconazole therapy. Currently, she was 8 months after surgery without evidence for recurrent disease.

## 2. Discussion

BA is characterized by the presence of an atretic bronchus that fails to communicate with the central airways. This typically occurs in the apicoposterior segment of the left upper lobe (as in our patient) but can also involve other lung areas [1, 2]. Patients are usually asymptomatic with imaging in late childhood or adulthood revealing a perihilar mass-like lesion (atretic, mucus filled dilated bronchus) along with distal emphysema in the area of lung supplied by that segmental or lobar bronchus. The emphysematous areas can show ectatic airways, mucocele, and signs of infection with air-fluid levels. The lung distal to the atretic bronchus is hyperinflated due to unidirectional ventilation occurring through intra-alveolar pores of Kohn and collateral communications from adjacent normal lung through canals of Lambert. The presence of a mass at the apex of a triangular hyperlucent lung parenchyma (as seen in our case, Figure 1) is highly suggestive of BA although nonpathognomonic. Bronchoceles are frequently seen due to retained secretions, “mucoid impaction sign,” which can appear as branched, linear, or spherical opacities [3, 4]. Bronchoscopy may show a blind-ending bronchus; however the chest CT scan remains the best imaging modality to diagnose BA and exclude other pathologies (especially tumors). During fetal development, bronchial buds start to develop around the fifth week of gestation, and BA likely occurs due to a vascular injury with subsequent focal ischemia of the developing bronchus. Alternatively, a separation of the bronchial bud can also occur during development. The latter theory may explain the reported association of BA with lung sequestration, bronchogenic cysts, and congenital lobar emphysema [5, 6]. BA has also been associated with nonpulmonary anomalies such as atrial septal defect, renal agenesis, pericardial defect, and left-sided inferior vena cava [3, 7, 8]. Pneumothorax may rarely develop secondary to rupture of the hyperinflated lung tissue adjacent to the atretic segment [9, 10]. Infections in the involved lung are infrequent due to the lack of direct communication with the rest of the tracheobronchial tree. Pneumonias can occur and are usually bacterial. Fungal infections are exceedingly rare in BA. In fact, although aspergillosis has been rarely reported in intralobar sequestration, to the best of our knowledge it has never been reported in BA [11, 12]. Our case is very unusual given the asymptomatic development of CPA after a latent period of more than 2 years from initial diagnosis. Periodic follow-up imaging may be beneficial in these patients even in the absence of symptoms. In summary, we report a unique case of chronic pulmonary aspergillosis in a patient with BA with associated lung emphysema.
